# Trends of Pandemic Parenting in Medical Academia

**DOI:** 10.5811/westjem.2022.6.54144

**Published:** 2022-09-15

**Authors:** Meeta Shah, Melissa Holmes, Inna Husain, Dayle Davenport, Sheila Dugan, Sobia Ansari

**Affiliations:** Rush Medical University Center, Department of Emergency Medicine, Chicago, Illinois

## Abstract

**Introduction:**

The pandemic has been difficult on physicians, with two fifths of doctors in one survey reporting that their mental health is now worse than before the pandemic. It is likely that a significant proportion of these physicians are parents of children necessitating childcare, as approximately 32% of the US workforce has someone in their household under the age of 14. We sought to study the impact of the coronavirus 2019 (COVID-19) pandemic on physician parents in academia. Our goal was to investigate the intersection of professional and personal challenges, as well as perceived impact on domestic life and professional development secondary to the COVID-19 pandemic.

**Methods:**

Using Survey Monkey, we developed a 37-question survey to address the aim of this study. Questions were grouped into four categories: demographics; impact on childcare; impact on care; and impact on mental health/wellness. Most of the questions were multiple choice with a few fill-in-the-blank options to allow participants to provide additional information related to their experiences as physicians during the pandemic. A link to the survey was disseminated via email to physicians at our home institution, Rush University Medical Center (Chicago), via our own intra- and interdepartmental communications, We used private social media accounts such as Facebook physician groups to reach out to physicians at other academic medical centers. Survey responses were voluntary and collected anonymously over an eight-week period, without identifiable data. Inclusion criteria included any physician identifying themselves as working full or full or part time in an academic facility in the US and caregivers for children <18 years.

**Results:**

Survey respondents were mostly female (83.2%), practicing in the Midwest (61.2%), and ranked as assistant professor (59.5%). The majority of respondents had two children (65.1%) who were <11 years in age (85.6%). Most respondents worked full time with 72.8% working over 50% clinically. Childcare was disrupted for 171 of 232 respondents (73.7%); 62.9% struggled with balancing work with childcare; 81.9% worried often or very often about fulfilling their responsibilities. A vast majority, 210 of 232 respondents (90.5%) had some degree of concern about feeling overburdened by their roles. More than half (57.3%) worried that their professional advancement was impacted by the pandemic, and 53.9% considered making adjustments to their clinical workload/. Over half (51.6%) thought that increased domestic responsibilities impacted their professional advancement.

**Conclusion:**

In the survey, which was completed primarily by early-career women physicians practicing in a variety of specialties and geographic regions, we noted that childcare disruption amidst the pandemic was extremely prevalent. The majority of respondents reported full-time equivalent work; thus, it is reasonable to assume that significant workloads and limitations in remote work in combination with childcare constraints resulted in significant burden. A large number felt the challenges were negatively impacting their professional development and felt overburdened by their various roles.

## INTRODUCTION

There are over half a million active physicians in the United States.^1^ The coronavirus 2019 (COVID-19) pandemic has been difficult for them, with two fifths of doctors in one survey reporting that their mental health is now worse than before the pandemic.^2^ Both anxiety and depression were found in over 22% of healthcare workers and insomnia in over 38%. Female healthcare workers exhibited higher rates of these symptoms compared to their male colleagues.^3^ It is likely that a significant proportion of these physicians are parents of children necessitating childcare, as approximately 32% of the US workforce has someone in their household <14 years old.^4^ The impact of the pandemic on parents, physicians or otherwise, has been significant. Due to large-scale school and daycare closures, as well as social distancing restrictions, many working parents were left without options for childcare and forced to balance their parental responsibilities with professional obligations.^5^ An October 2020 survey-based study found that since March 2020, almost a quarter of parents reported worsening mental health, and 14% reported worsening behavioral issues for their children.^5^

While the impacts of the COVID-19 pandemic have been far reaching, we sought to study its impact specifically on physician parents in academia, irrespective of gender. Our goal was to investigate the burden of professional and personal obligations. We also surveyed their perceived professional and personal challenges secondary to the COVID-19 pandemic, as a result of the increased intersection between work and domestic roles. Finally, we sought to inquire about their mental health challenges and any changed perceptions of value in their professional careers.

## METHODS

### Study Design and Participants

We developed a survey consisting of 37 questions, using SurveyMonkey (Momentive, San Mateo, CA). The questions were grouped into four categories: demographics; impact on childcare; impact on career; and impact on mental health/wellness. The first 12 questions focused on demographics, including the survey participants’ gender, number of children and their ages, profession and academic position, marital status, and geographic location. The seven questions in the second category – impact on childcare – focused on survey participants’ baseline childcare requirements, changes brought on due the pandemic, and challenges in balancing professional and childcare duties. The 10 questions in the third category – impact on career – focused on changes in workload, ability to meet professional responsibilities, and perceived impact on professional advancement. The final category included eight questions focused on mental and physical stressors related to working as a physician during the COVID-19 pandemic. Most of the questions were multiple-choice format with a few fill-in-the-blank options to allow participants to provide additional information related to their experiences as physicians during the pandemic.

**Population Health Research Capsule**

What do we already know about this issue?

*Two-fifths of doctors in one survey reported worse mental health than pre-pandemic Female healthcare workers exhibited higher rates of anxiety and depression compared to male colleagues*.

What was the research question?

*We sought to investigate mental health challenges and perceptions of value in career for parents in academia due to the pandemic*.

What was the major finding of the study?

*Childcare was disrupted for 73.7%, 81.9% worried about fulfilling responsibilities and 90% felt overburdened by personal and professional roles*.

How does this improve population health?

*Mental health stressors, prevalent in this cohort, could impact quality of care, safety, and clinical outcomes and reduced engagement, which in turn could impact professional advancement*.

### Data Collection

A link to the survey was disseminated via email to physicians at our home institution, Rush University Medical Center (Chicago), via intra- and interdepartmental communications. We used private social media accounts such as Facebook physician groups to reach out to physicians at other academic medical centers. Inclusion criteria for participation in the survey study included academic physicians who were also parents working in the US and able to self-administer the survey. Survey responses were voluntary and collected anonymously via SurveyMonkey over an eight-week period, without identifiable data. Responses were password protected with only study investigators having access to the data collected. Inclusion criteria included any physician identifying themselves as working full or part time in an academic facility in the US and caregivers for children <18 years.

## RESULTS

Survey respondents were mostly female (83.2%), practicing in the Midwest (61.2%), and ranked as assistant professor (59.5%). The majority of respondents had two children (65.1%) who were <11 years in age (85.6%) and did not have special needs (89.2%). Most respondents had a consistent partner to assist in childcare (94.8%) and worked 81–100% total full-time equivalent (FTE) (83.2%) with 72.8% working over 50% clinically; 108 out of 232 respondents (46.65%) were dual physician households. Physicians from 26 specialties responded, with the top five representing pediatrics, emergency medicine, internal medicine, family medicine, and otolaryngology (Table 1).

### Domestic Duties and Well-being

**Figure 1:**
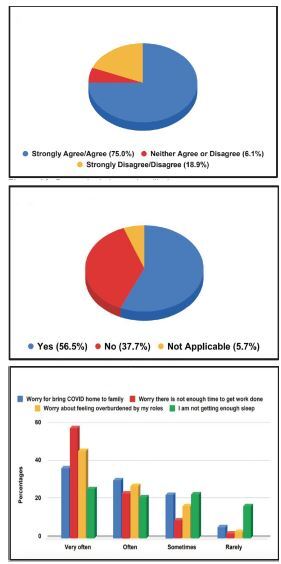
Domestic duties and wellbeing of survey respondents. (A) Respondents who experienced a disruption in childcare. (B) Respondents who experienced difficulties securing childcare. (C) Respondents who experienced indicators of physician burnout.

During the pandemic, childcare was disrupted for 171 of 232 respondents (73.7%) and difficult to secure or maintain for 129 respondents (56.6%) (Figures 1A, 1B). A total of 62.9% struggled with balancing work with childcare, with 21.6% taking on most of the new childcare responsibilities themselves and 25% splitting new responsibilities with their spouse/partner. We found that 81.9% of respondents worried often or very often”about fulfilling their responsibilities; and 210 of 232 respondents (90.5%) expressed some degree of concern about feeling overburdened by their roles, with 47.4% endorsing often or very often not getting enough sleep (Figure 1C). About 41% said that challenges with childcare have had a negative impact on their ability to perform their jobs. An overwhelming 87% of respondents expressed some concern about exposing immediate family members to COVID-19 (Figure 1C).

### Professional Impact

**Figure 2:**
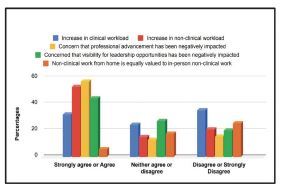
Professional impact of the COVID-19 pandemic.

Survey participants were mixed in their responses to how their clinical workload was affected by the pandemic. When asked whether their clinical workload had increased, 35% disagreed or strongly disagreed, 32.3% agreed or strongly agreed, and 24.6% neither agreed or disagreed. More than half of respondents (53.55%) thought their non-clinical workload increased, and 37.9% disagreed or strongly disagreed that non-clinical work completed from home was equally valued to in-person work. More than half (57.3%) worried that their professional advancement was impacted by the pandemic (Figure 2), and 53.9% considered making adjustments to their clinical workload/FTE (Table 2). Of the respondents, 44.4% worried that their visibility for leadership opportunities had been impacted, and over half (51.6%) thought that increased domestic responsibilities impacted their professional advancement.

## DISCUSSION

In this survey study completed primarily by early-career women physicians practicing in a variety of specialties and geographic regions, we noted that childcare disruption amidst the pandemic was extremely prevalent and about half of all respondents endorsed difficulty securing childcare, in line with previous studies during the pandemic.^6^ Considering that the majority of our respondents reported full-time work, it is reasonable to assume that significant clinical workloads, limitations in remote work, and childcare constraints (including remote learning environments) resulted in significant burden. Worrying about the exposure of COVID-19 to immediate family members seemed to be an additional stressor.

It is likely that dual-earner couples had to determine how to take on extra childcare responsibilities based on their employment situation and family dynamics. Pre-pandemic data illustrated that one working spouse (usually the female) did the majority of childcare and more housework in the family.^7^ In a study of young physician-researchers of married or partnered respondents with children, women spent 8.5 more hours per week on domestic activities. For those with partners employed full time, women were more likely to take time off during disruptions of usual childcare arrangements than men.^8^ The concern here again is that women may have been increasingly more impacted in these family units based on historic data and our survey findings.

With regard to certain social and economic factors, working women have been affected more than men during this pandemic. For example, women are more at risk of unemployment,^9^ and for the first time one in four women considered stepping out of the workforce or downshifting their careers, with women in senior roles, working mothers, and women of color most at risk.^10^ The outcome of this trend has also been studied by Edwards, who found that women, parents, and women who are parents would leave the workforce in rising order, resulting in the loss of significant wages.^11^ Considering the findings of these prior studies, the responses of the parents in our survey, and the fact that individuals identifying as female make up 37% of US physicians, there is significant concern for ongoing deleterious impacts on this group.^12^

Many participants also noted increased factors in burnout, clinical or non-clinical workloads (Figure 2). Almost half reported poor sleep and felt their work was negatively impacted. These mental health stressors could lead to reduced quality of care, safety, and clinical outcomes for patients^13^ and reduced engagement, which in turn could impact professional advancement and leadership opportunities. The perception that the pandemic had negatively impacted professional development was a concern for the majority of our respondents, which we found alarming. With approximately 30% of healthcare workers having had thoughts of leaving the profession during the pandemic,^14^ it is imperative that institutions and federal agencies address the short- and long-term impacts of the COVID-19 pandemic on this cohort.

This study provides insights into how physicians, in particular women and parents, experienced the COVID-19 pandemic in the US during the first wave. Future studies could assess the immediate and long-term impacts in the personal and professional arenas as well as impacts on the healthcare system at large including physician availability, wellness and retention.

## LIMITATIONS

We acknowledge certain limitations in our study. There were only 232 respondents, and the majority were female and from non-surgical specialties. A higher response rate with more survey responses from male physicians and physicians from surgical specialties would improve generalizability. Second, the survey was distributed in June when most children were not in school. Responses related to childcare stressors might have been different if the survey were performed during the school year and parents were actively trying to home-school or balance school year activities with professional responsibilities.

## CONCLUSION

We found that childcare disruption amidst the pandemic was prevalent among early-career women physicians, most of whom practiced full time in a variety of specialties and geographic regions. These findings led us to conclude that significant workloads and limitations in remote work, in combination with childcare constraints, resulted in significant burden. A large number of survey respondents felt the challenges were negatively impacting their professional development and that they felt overburdened by their various roles.

## Supplementary Information



## Figures and Tables

**Table 1:** Demographic Data of Survey Respondents.

Total respondents	232	
Number of children		
1	33	14.2%
2	151	65.1%
3	41	17.7%
4 or more	7	3.0%
Age of children		
0–4	152	-
5–7	105	-
8–11	71	-
12–15	30	-
16–19	18	-
19+	7	-
Providers with children who have special needs		
Yes	25	10.8%
No	207	89.2%
Partner in childcare		
Yes	220	94.8%
Partial	3	1.3%
No	9	3.9%
Academic title		
Instructor	28	12.1%
Assistant Professor	138	59.5%
Associate Professor	40	17.2%
Professor	6	2.6%
Other	20	8.6%
Current total full time equivalents (FTE)		
81 to 100%	193	83.2%
51 to 80%	33	14.2%
0 to 50%	6	2.6%
Clinical specific FTE		
76 to 100%	103	44.4%
51 to 75%	67	28.9%
26 to 50%	39	16.8%
0 to 25%	23	9.9%
Gender		
Male	38	16.4%
Female	193	83.2%
Other/ not listed	1	0.4%
Geographical areas		
Midwest	142	61.2%
South	43	18.5%
Northeast	31	13.4%
West	16	6.9%
Top 5 reporting specialties		
Internal Medicine	23	9.9%
Pediatrics	59	25.4%
Family Medicine	21	9.1%
Emergency Medicine	39	16.8%
Otolaryngology	13	5.6%
Total of these specialties	155	66.8%
Dual physician households		
Respondents with 2 physician households	108	46.6%
Top 5 childcare arrangements		
Full time school with or without after school activities	97	41.8%
Full time nanny or au pair	82	35.3%
Full time daycare	67	28.9%
External family member assisting in childcare	42	18.1%
Part time nanny	30	12.9%

**Table 2:** Professional impact of the COVID-19 pandemic.

Considerations of Professional Shifts in Medicine Due to Pandemic		
In the past 4 months I have considered making adjustments to my clinical workload/FTE		
Yes	125	53.9%
Factors impacting worry about professional advancement		
Increased domestic responsibilities	113	48.7%
Home-schooling requirements	89	38.4%
Increased use of virtual meeting platforms	76	32.8%
Inability to do non-clinical work from home	61	26.3%
Pandemics Affect on Professional Advancement		
Those who strongly agree or agree that the pandemic has negatively impacted their professional advancement.	133	57.3%

*FTE*, Full-time equivalent.
