# Supplementary material for: Trends of Pandemic Parenting in Medical Academia
Source: West J Emerg Med. 2022 Sep 15;23(5):678–83. doi: 10.5811/westjem.2022.6.54144 (PMC9541971; doi:10.5811/westjem.2022.6.54144)
Supplement: Supplementary file 1 [file wjem-23-678-s001.pdf]

RUSH UNIVERSITY

Pandemic Parenting in Medical Academia

1.

This is a survey developed by a group of physicians at Rush University Medical Center on the impact of the COVID-19 pandemic on physician parents in academia (previous, current and aftermath). The responses to this survey will be reported anonymously and will be part of an IRB exempt study with an intent to be submitted for publication. Please only complete the survey once.
(Note: The multiple choice answers are required to proceed. If you skip any of the optional questions, the survey tool will have you review your answers when you click "next", so pressing "next" again will get you to the next page).

Dr. Sobia Ansari
Dr. Dayle Davenport
Dr. Sheila Dugan
Dr. Inna Husain
Dr. Melissa Holmes
Dr. Meeta Shah

* 1. How many children do you have?

- ○ 1
- ○ 2
- ○ 3
- ○ 4 or more

* 2. What age(s) are your children (select all that apply):

- ☐ 0 to 4
- ☐ 5 to 7
- ☐ 8 to 11
- ☐ 12 to 15
- ☐ 16 to 19
- ☐ Over 19

* 3. Do you have any children with special needs (ie. particular educational requirements resulting from learning difficulties, physical disability, or emotional and behavioral difficulties.)

- Yes
- No

* 4. Do you have a consistent partner to assist in Childcare?

- Yes - Spouse, Domestic Partnership or Live in Partner
- Partial - Divorced (shared custody), In a Relationship but not living together, Other
- No - Single, Widowed, Other

* 5. If married, part of a domestic partnership or have a live in partner: are you part of a dual physician household?

- Yes
- No
- Not Applicable (N/A)

* 6. What is your academic title?

- Clinical Instructor
- Assistant Professor
- Associate Professor
- Professor
- Other

* 7. What is your current **TOTAL** FTE (full-time equivalent) ***- both clinical and non-clinical total?***

- 0 to 50%
- 51 to 80%
- 81 to 100%

* 8. What is your current **CLINICAL** FTE?

- 0 to 25%
- 26 to 50%
- 51 to 75%
- 76 to 100%

* 9. What is your current **NON-CLINICAL** (education, administrative, research) FTE?

- ◯ 0 to 25%
- ◯ 26 to 50%
- ◯ 51 to 75%
- ◯ 76 to 100%

* 10. What is your specialty (select all that apply)?

- [ ] Allergy and Immunology
- [ ] Anesthesia
- [ ] Cardiology
- [ ] Dermatology
- [ ] Emergency Medicine
- [ ] Endocrine
- [ ] Family Medicine
- [ ] Gastroenterology
- [ ] Hematology Oncology
- [ ] Infectious Disease
- [ ] Internal Medicine
- [ ] Nephrology
- [ ] Neurology
- [ ] Obstetrics and Gynecology
- [ ] Ophthalmology
- [ ] Orthopedics
- [ ] Otolaryngology
- [ ] Pediatrics
- [ ] Physical Medicine and Rehabilitation
- [ ] Plastic Surgery
- [ ] Psychiatry
- [ ] Pulmonary/Critical Care
- [ ] Radiology
- [ ] Rheumatology
- [ ] Surgery (General and Subspecialty)
- [ ] Urology
- [ ] OTHER

* 11. To which gender do you most identify?

- Male
- Female
- Non-Binary
- Not listed or other

* 12. Please identify your general geographic area within the United States

- Midwest (Ohio, Michigan, Indiana, Wisconsin, Illinois, Minnesota, Iowa, Missouri, North Dakota, South Dakota, Nebraska, and Kansas.)
- Northeast (Maine, New Hampshire, Vermont, Massachusetts, Rhode Island, Connecticut, New York, New Jersey, and Pennsylvania.)
- South (Delaware, Maryland, Virginia, West Virginia, Kentucky, North Carolina, South Carolina, Tennessee, Georgia, Florida, Alabama, Mississippi, Arkansas, Louisiana, Texas, and Oklahoma)
- West (Montana, Idaho, Wyoming, Colorado, New Mexico, Arizona, Utah, Nevada, California, Oregon, Washington, Alaska, and Hawaii)

RUSH UNIVERSITY

Pandemic Parenting in Medical Academia

2. Impact on Childcare

* 13. Prior to the pandemic, my childcare arrangement included (select all that apply):

- [ ] Full time nanny or Au Pair
- [ ] Part time nanny
- [ ] Full time daycare
- [ ] Part time daycare
- [ ] External family member assisting in childcare
- [ ] Splitting childcare with partner/spouse
- [ ] Full time school with or without after school activities
- [ ] Children are old enough to be home independently
- [ ] Other

* 14. My childcare arrangement(s) was/were interrupted during the pandemic?

- ○ Strongly agree
- ○ Agree
- ○ Neither agree nor disagree
- ○ Disagree
- ○ Strongly disagree

* 15. I had difficulty finding/securing/maintaining childcare?

- ○ Yes
- ○ No
- ○ N/A

* 16. If you selected **"YES"** to question 15, select all that apply to you.

(If you replied "no" to question 15 please choose one of the two last selections below.)

- [ ] This had a negative impact on my ability to do my job
- [ ] I had to take on most of the new childcare responsibilities
- [ ] My spouse/partner took on most of the new childcare responsibilities
- [ ] My spouse/partner and I divided the new childcare responsibilities
- [ ] I had to cut back at work to accommodate childcare responsibilities
- [ ] I had to resign from my position or particular roles to accommodate childcare responsibilities
- [ ] This had no impact on my ability to do my job
- [ ] Not applicable (answered no to question 13)

* 17. I am currently struggling with balancing work with childcare

- ( ) Strongly agree
- ( ) Agree
- ( ) Neither agree nor disagree
- ( ) Disagree
- ( ) Strongly disagree

* 18. I am worried about balancing work with childcare if schools or daycares remain closed this fall

- ( ) Strongly agree
- ( ) Agree
- ( ) Neither agree nor disagree
- ( ) Disagree
- ( ) Strongly disagree
- ( ) Not applicable

19. Is there anything else you would like to add regarding the impact of the pandemic on childcare?

RUSH UNIVERSITY

Pandemic Parenting in Medical Academia

3. Impact on career

* 20. My clinical workload increased during the pandemic

- Strongly agree
- Agree
- Neither agree nor disagree
- Disagree
- Strongly disagree
- Not applicable

* 21. My non-clinical workload increased during the pandemic

- Strongly agree
- Agree
- Neither agree nor disagree
- Disagree
- Strongly disagree
- Not applicable

* 22. I am able to complete my non-clinical work from home

- Strongly agree
- Agree
- Neither agree nor disagree
- Disagree
- Strongly disagree
- Not applicable

* 23. I am worried that my professional advancement is negatively impacted by the pandemic

- Strongly agree
- Agree
- Neither agree nor disagree
- Disagree
- Strongly disagree

* 24. **If you answered yes to question 23, please select all that apply: I am worried that my professional advancement is negatively impacted by the pandemic due to:**

(please answer N/A if you answered "No" to question 23):

- Increased domestic responsibilities
- Home-schooling requirements
- Increased use of virtual meeting platforms such as WebEx and Zoom
- Inability to do non-clinical work from home
- The pandemic has not negatively impacted my professional advancement
- Other
- Not applicable

25. If you chose "Other" for question 24 would you like to elaborate?

* 26. I have worried that my visibility for leadership opportunities has been negatively impacted by the pandemic

- Strongly agree
- Agree
- Neither agree nor disagree
- Disagree
- Strongly disagree
- Not applicable

* 27. I feel that my non-clinical work completed from home is equally valued to in-person work by my institution and department

- Strongly agree
- Agree
- Neither agree nor disagree
- Disagree
- Strongly disagree
- Not applicable

* 28. In the last 4 months, I have considered making adjustments to my clinical workload/FTE due to the pandemic

- Yes
- No

29. Is there anything else you would like to add regarding the impact of the pandemic on career development?

RUSH UNIVERSITY

Pandemic Parenting in Medical Academia

4. Impact on mental health/self care due to the pandemic

Please answer the following questions specifically with relation to how the pandemic has impacted you

* 30. I worry about job security

- ◯ Very often
- ◯ Often
- ◯ Sometimes
- ◯ Rarely
- ◯ Never

* 31. I worry about bringing COVID home to my family

- ◯ Very Often
- ◯ Often
- ◯ Sometimes
- ◯ Rarely
- ◯ Never

* 32. I worry about not having enough time to get everything done

- ◯ Very Often
- ◯ Often
- ◯ Sometimes
- ◯ Rarely
- ◯ Never

* 33. I worry about feeling overburdened by my roles

- Very often
- Often
- Sometimes
- Rarely
- Never

* 34. I worry about needing to cancel or reschedule professional duties

- Very Often
- Often
- Sometimes
- Rarely
- Never

* 35. I am not getting enough sleep (or am sleeping less than I used to) specifically secondary to the impacts of the pandemic

- Very Often
- Often
- Sometimes
- Rarely
- Never

36. Is there anything else you would like to add regarding the impact of the pandemic on your mental health or self-care?

37. Optional: If you feel comfortable, please list where you are employed so that aggregate data may be sent to your women's leadership/affinity group if it is possible. This information is anonymous and will be used only to identify institutional specific concerns.

RUSH UNIVERSITY

Pandemic Parenting in Medical Academia

5. Thank you!

Thank you for taking this survey, we appreciate the time you took out to provide us further information on the impact of the pandemic for parents who are in medical academia.

Dr. Sobia Ansari
Dr. Dayle Davenport
Dr. Sheila Dugan
Dr. Inna Husain
Dr. Melissa Holmes
Dr. Meeta Shah
